# A comparison of the clinical efficacy of GON block at the C2 level and GON block at the classical distal occipital level in the treatment of cluster headache

**DOI:** 10.22514/jofph.2025.080

**Published:** 2025-12-12

**Authors:** Anıl Kılınç, Mustafa Karaoğlan

**Affiliations:** ^1^Department of Anesthesia and Reanimation, Ordu University, 52100 Ordu, Turkey; ^2^Department of Algology, Ordu State Hospital, 52100 Ordu, Turkey

**Keywords:** C2 GON block, GON block, Cluster headache, Greater occipital nerve, Distal GON block

## Abstract

**Background**: This study aimed to compare the clinical efficacy of C2-level and distal greater occipital nerve (GON) blockade in the management of cluster headache (CH), focusing on pain relief onset and overall treatment outcomes. **Methods**: In this retrospective study, 48 patients diagnosed with CH were divided into two equal groups: one receiving bilateral C2 GON blocks and the other undergoing bilateral distal GON blocks. Both groups were treated with dexamethasone as the steroid and bupivacaine as the local anaesthetic. Pain relief and treatment duration were assessed, and adverse events were monitored. **Results**: Both GON blockade techniques were effective in reducing CH symptoms. Patients treated with C2 GON blocks achieved complete pain relief by the third week, allowing for treatment cessation. In contrast, the distal GON block group required up to six injections for similar levels of relief. Rapid pain control was more pronounced in the C2 GON group, with no significant adverse effects observed in either group. **Conclusions**: C2 GON blockade demonstrated greater efficacy for early pain relief in CH patients compared with distal GON blockade. These findings suggest that C2 GON blockade may be a more suitable option for both transitional and preventive therapy in CH. Further studies are recommended to confirm these results and to explore the long-term benefits of C2 GON blockade in CH management.

## 1. Introduction

Cluster headache (CH) is a rare but significant type of primary headache, classified under trigeminal-autonomic cephalalgias (TACs) according to the International Classification of Headache Disorders, 3rd edition (ICHD-3) [1, 2]. While it is less common than conditions such as migraine or tension-type headache, CH still affects approximately one in 500 people globally, a prevalence similar to that of multiple sclerosis [3]. Those who suffer from CH endure excruciating, unilateral headache attacks that can last between 15 and 180 minutes, occurring up to eight times per day. These attacks are often accompanied by ipsilateral cranial autonomic symptoms or feelings of restlessness, or both.

CH generally manifests in two clinical forms:

Episodic CH, with cluster periods typically lasting weeks or months and remission periods of at least three months, and Chronic CH, with attacks persisting for more than 12 weeks without remission or with remissions lasting less than three months [3].

Despite its relatively low prevalence, CH poses a serious health concern due to the extreme pain it causes and its association with elevated suicidal risk [4].

Medical treatment strategies for CH focus on three main approaches: abortive (acute) therapies, designed to quickly alleviate symptoms during an attack; preventive therapies, aimed at reducing the frequency and severity of attacks over time; and transitional therapies, which serve as a temporary or bridging solution to manage attacks while waiting for preventive treatments to take full effect [5]. Acute treatments, such as subcutaneous sumatriptan or high-flow oxygen, are administered at the onset of an attack to terminate an acute episode, whereas preventive therapies, such as verapamil or lithium, aim to decrease the intensity, duration, or frequency of future attacks. Transitional therapy most commonly involves oral corticosteroids [5], though their prolonged use is associated with significant adverse effects, and abrupt discontinuation may trigger a rebound of headache attacks.

A viable alternative transitional therapy is the greater occipital nerve block (GONB), involving suboccipital injections of steroids, local anesthetics, or both. The effectiveness of a single GONB for episodic CH has been demonstrated in both open-label and placebo-controlled studies [6].

The C2 GON block is a more proximal fascial block that allows the administration of a larger volume of solution. Studies in chronic migraine have compared this technique with the distal approach, reporting potential advantages such as prolonged duration of effect and reduced cosmetic side effects [7]. However, to date, no studies have directly compared C2 GON and distal GON blocks in the treatment of CH. In this study, we hypothesized that the C2 GON block, due to its proximal application, might avoid the cosmetic side effects potentially associated with the distal approach when using steroid solutions and may provide enhanced efficacy through the use of a greater injectate volume.

## 2. Materials and methods

### 2.1 Participants and methods

This retrospective study was conducted at the Pain Center of Ordu State Hospital, Ordu, Turkey, using data from headache diaries collected between September 2021 and August 2024. Diagnoses of CH were confirmed according to the International Classification of Headache Disorders, 3rd Edition (ICHD-3) criteria [2]. The episodic nature of CH in these patients, characterized by cluster periods typically lasting 1–3 months, was consistent with the diagnostic definition.

Inclusion criteria were:

• Age between 18 and 70 years;

• Symptom onset within the preceding month;

• Diagnosis of episodic CH per ICHD-3 criteria;

• Completion of either bilateral C2 greater occipital nerve (C2 GON) blocks or bilateral distal occipital GON (DOGON) blocks as part of treatment;

• Availability of complete headache diary data before and after treatment.

Exclusion criteria were:

• Incomplete headache diaries or missing treatment records;

• Refusal to allow pseudonymized data to be used for research purposes;

• Contraindications to injection therapy (infection at the injection site, coagulation disorders, pregnancy, or prior surgery in the occipital region).

Patients were categorized based on the treatment received: 24 underwent bilateral C2 GON blocks and 24 received bilateral DOGON blocks.

Treatment outcomes were self-reported and classified as:

• Clinical relief: reduction in headache severity to a Visual Analog Scale (VAS) score <4 and at least a 50% reduction from baseline pain intensity;

• Complete painlessness: total absence of headache (VAS = 0).

### 2.2 Procedure and intervention

#### 2.2.1 Distal occipital GON block (DOGON)

The DOGON protocol was performed as previously described [8]. Patients were positioned sitting or prone, and the external occipital protuberance was palpated. Bilateral injections were made 2 cm lateral and 2 cm inferior to the protuberance after antiseptic preparation. Each injection consisted of 1.5 mL per side prepared with 1 mL 0.5% bupivacaine plus 0.5 mL (2 mg dexamethasone), for a total bilateral volume of 3 mL per session. A 22-gauge spinal needle was used. Treatment was administered weekly for up to four weeks, with a 30-minute post-procedure observation period.

#### 2.2.2 C2 GON block

The C2 GON block was performed according to the ultrasound-guided method described in [9]. Patients were positioned prone with the neck flexed. A 12–18 MHz linear probe was placed transversely over the occipital prominence and moved caudally to visualize C1 and C2 landmarks, including the obliquus capitis inferior (OCI) and semispinalis capitis (SSC) muscles. The target was identified between the OCI and SSC, avoiding the occipital artery (Fig. 1).

**Fig. 1.** Greater occipital nerve block at the level of C2 with ultrasound guided. (A) Anatomical illustration of GON block performed at the C2 level. (B) Corresponding ultrasound image of the same view. GON: Greater occipital nerve; TON: Third occipital nerve; OCI: Obliquis capitis inferior muscle; C2 SP: Spinal process of C2.

Due to the close proximity to the vertebral artery, 0.5% bupivacaine was diluted with normal saline to obtain a final concentration of 0.025%, in order to minimize the risk of local anesthetic toxicity while maintaining adequate injectate volume for nerve coverage. Under ultrasound guidance, 4 mL per side (final concentration 0.025% bupivacaine combined with 2 mg dexamethasone) was injected, for a total bilateral volume of 8 mL per session.

All injections in both groups were performed by the same pain specialist to ensure procedural consistency.

### 2.3 Statistical analysis

Baseline demographic and clinical characteristics were summarized using descriptive statistics. Categorical variables were expressed as counts and percentages; continuous variables as mean ± standard deviation (SD); and non-normally distributed variables as median (interquartile range (IQR)).

Between-group comparisons for categorical variables were conducted using Chi-square or Fisher’s exact tests. For continuous variables, independent-sample *t*-tests or Mann-Whitney U tests were applied depending on normality. Statistical comparisons were performed both for the primary tables (Tables 1 and 2) and for **Supplementary Tables 1,2** and Table 3 containing additional pain intensity change data, as requested by the reviewer.

**Table 1.** Demographic characteristics and medication use of patients receiving C2 GON and distal GON blocks.

Parameter		Total (n = 48)	C2 GON (n = 24)	Distal GON (n = 24)
Number of injections		137	37	100
	Initial injections	48	24	24
	Repeated injections (total)	89	13	76
Male, n (%)		37 (77.1%)	19 (79.2%)	18 (75.0%)
Age, mean ± SD (yr)		37.23 ± 5.20	38.17 ± 5.41	36.29 ± 5.02
Weekly attack frequency, median (IQR)		20.5 (18.0–26.5)	20.0 (16.5–27.3)	20.5 (18.3–26.5)
Prophylactic medication, n (%)				
	None	4 (8.3%)	3 (12.5%)	1 (4.2%)
	At least one medication	44 (91.7%)	21 (87.5%)	23 (95.8%)
	-Verapamil	13 (27.1%)	6 (25.0%)	7 (29.2%)
	-Topiramate	32 (66.7%)	15 (62.5%)	17 (70.8%)
	-Prednisolone	0 (0%)	0 (0%)	0 (0%)
	-Lithium	0 (0%)	0 (0%)	0 (0%)
Acute medication, n (%)				
	Frovatriptan	20 (41.7%)	10 (41.7%)	10 (41.7%)
	Rizatriptan	28 (58.3%)	14 (58.3%)	14 (58.3%)
	Oxygen	16 (33.3%)	7 (29.2%)	9 (37.5%)

*GON: greater occipital nerve; SD: standard deviation; IQR: interquartile range.*

**Table 2.** C2 GON and distal GON weekly application numbers and distribution of clinical relief and complete painlessness data with statistical comparison.

Week	Total	C2 GON (n)	Distal GON (n)	*p*-value (test)	Complete Painlessness n (%)	*p*-value (test)
First GON Blockage	48	24	24			
Clinical Relief	28	20 (83%)	8 (33%)	0.0013 (Chi-square)	15 (62%)	0.0003 (Chi-square)
Complete Painlessness	17	15 (62%)	2 (8%)			
Second GON Blockage	31	9	22			
Clinical Relief	15	9 (100%)	6 (27%)	0.0002 (Fisher’s Exact)	5 (55%)	0.0039 (Fisher’s Exact)
Complete Painlessness	6	5 (55%)	1 (4%)			
Third GON Blockage	27	4	23			
Clinical Relief	14	4 (100%)	10 (43%)	0.0978 (Fisher’s Exact)	4 (100%)	0.0040 (Fisher’s Exact)
Complete Painlessness	8	4 (100%)	4 (17%)			
Fourth GON Blockage	19	-	19		10 (52%)	
Clinical Relief	10	-	10 (52%)			
Complete Painlessness	9	-	9 (47%)			
Fifth GON Blockage	10	-	10		8 (80%)	
Clinical Relief	8	-	8 (80%)			
Complete Painlessness	8	-	8 (80%)			
Sixth GON Blockage	2	-	2		2 (100%)	
Clinical Relief	2	-	2 (100%)			
Complete Painlessness	2	-	2 (100%)			

*GON: Greater Occipital Nerve.*

**Table 3.** Weekly clinical relief and complete painlessness rates with statistical comparisons.

Week	Outcome	C2 GON % (n/N)	Distal GON % (n/N)	Test statistic	*p*-value	Effect size (φ)
1	Clinical relief	83% (20/24)	33% (8/24)	χ^2^ (1, N = 48) = 10.49	0.0013	0.47
1	Complete painlessness	62% (15/24)	8% (2/24)	χ^2^ (1, N = 48) = 13.17	0.0003	0.52
2	Clinical relief	100% (9/9)	27% (6/22)	Fisher’s exact	0.0002	0.74
2	Complete painlessness	55% (5/9)	4% (1/22)	Fisher’s exact	0.0039	0.52
3	Clinical relief	100% (4/4)	43% (10/23)	Fisher’s exact	0.0978	0.37
3	Complete painlessness	100% (4/4)	17% (4/23)	Fisher’s exact	0.0040	0.53
4	Clinical relief	-	52% (10/19)	NA	NA	NA
4	Complete painlessness	-	47% (9/19)	NA	NA	NA
5	Clinical relief	-	80.0% (8/10)	NA	NA	NA
5	Complete painlessness	-	80.0% (8/10)	NA	NA	NA
6	Clinical relief	-	100% (2/2)	NA	NA	NA
6	Complete painlessness	-	100% (2/2)	NA	NA	NA

*GON: Greater Occipital Nerve.*

A *p*-value < 0.05 was considered statistically significant.

## 3. Results

A total of 48 patients were included in the analysis. In this study, 24 patients received C2 GON injections, while 24 patients underwent distal GON injections. Baseline demographic and clinical characteristics, including sex distribution, mean age, weekly attack frequency, prophylactic medication use, and acute medication use, are presented in Table 1. No statistically significant differences were found between the two groups for these variables, as shown in **Supplementary Table 1**, which reports the inferential statistics and effect sizes for each baseline comparison. These results indicate that the treatment groups were comparable and that any observed differences in outcomes are unlikely to be attributable to baseline imbalances.

Over the treatment period, a total of 137 injections were administered: 48 initial and 89 repeated injections. The C2 GON group received fewer total injections (n = 37) compared to the distal GON group (n = 100), reflecting the faster achievement of pain control in the former (Table 2). The between-group statistical comparison, presented in **Supplementary Table 2**, confirmed that this difference was statistically significant with a large effect size.

Weekly treatment outcomes in terms of clinical relief and complete painlessness rates for each group are detailed in Table 3. During the first two weeks, the C2 GON group demonstrated significantly higher rates of both clinical relief and complete painlessness compared to the distal GON group. By week 3, the difference in clinical relief was no longer statistically significant, although complete painlessness remained significantly higher in the C2 GON group. Only distal GON patients required injections beyond week 3, with gradual improvement observed until all patients achieved complete painlessness by week 6.

These findings demonstrate that C2 GON blocks offer superior early clinical efficacy compared to distal GON blocks, achieving rapid pain control with fewer injections. Distal GON blocks eventually reached comparable pain control rates, but only after repeated weekly applications extending to the sixth week (Fig. 2).

**Fig. 2.** Clinical relief and complete painlessness percentages by week. GON: greater occipital nerve.

## 4. Discussion

In our study, a cohort of 48 patients diagnosed with CH reported significant relief by the end of the sixth week following GON block treatment (Fig. 3). In the group of 24 patients who received distal level GON block, rapid control of attack frequency and severity was not sufficiently achieved; however, significant improvement occurred after the third injection, and complete relief was achieved by the sixth injection. In contrast, in the other group of 24 patients treated with C2 GON block, relief began earlier, and all patients achieved complete relief by the end of the third week, at which point treatment was discontinued. Although previous studies have documented the efficacy of C2 level GON block in patients with chronic migraine, we could not find any literature specifically addressing its application in the treatment of CH [10, 11, 12].

**Fig. 3.** Follow-up diagram of study. GON: greater occipital nerve.

The distal GON block technique has been well-documented in the literature; however, there has been variability in the local anaesthetics and steroids employed. Due to the arterial proximity and close association with direct cranial vessels in the C2 GON block, the use of dexamethasone, a particulate-free steroid, was deemed necessary to prevent serious complications. The use of particulate steroids in such cases carries a risk of inadvertent intravascular injection, which could lead to vertebral artery occlusion and result in severe outcomes such as stroke or paralysis [13]. Therefore, in our study, we administered bupivacaine as the most commonly used local anaesthetic alongside dexamethasone as the steroid. We utilized the same local anaesthetic and steroid for the distal GON block to ensure there were no differences for this study [9].

We identified several distinctions in our findings compared with other studies in the literature involving patients with CH treated with distal GON blockade. Although the responder rate in the distal GON group was initially 33.33% at the end of the first week, this early finding should not be directly compared to the efficacy rates reported in the literature without considering the timing and total duration of treatment. In our study, the rate of clinically relevant improvement in the distal GON group rose to 43.48% by the end of the fourth week. This value approaches the lower bound (47.8%) of response rates typically reported in previous reviews [14, 15]. Most published studies recommend a GON block regimen lasting 3 to 4 weeks, and their efficacy evaluations are generally based on follow-ups conducted between 10 and 30 days post-treatment [15, 16].

Although our study continued GONB treatment up to six weeks in some patients, we chose to focus on the fourth-week outcomes for comparison purposes, as this time point represents the most reported endpoint in the literature and reflects a standardized evaluation window. Both our study and prior reviews emphasize that the fourth week serves as a practical and clinically relevant benchmark for assessing treatment response [14, 15]. The relatively lower response observed in our study for the distal GON group compared to some prior reports for patients with CH may be a result of variability in headache severity and natural fluctuations inherent to the episodic cluster headache population. Nevertheless, the observed difference remains consistent with the range of outcomes reported in the existing literature.

Even in primary headaches like chronic migraine, where pain intensity is generally lower than in CH, complete relief was rarely achieved with a single distal GON blockade. Despite differing pathogenesis, repeated injections were generally essential for effective pain management with distal GON blockade [17]. Although comparative studies between C2 and distal GON blockade for chronic migraine showed no significant differences in outcomes at the 3-month follow-up, study reports indicated that C2-level GON blockade provided a faster onset of pain relief in the short term [7, 18].

In a previous study we conducted comparing C2 GON blockade with distal GON blockade techniques for the treatment of chronic migraine, we did not observe a significant difference in efficacy between the two approaches [7]. However, in this study, we found a significant difference in treatment effectiveness for patients with CH. Based on our results and prior literature, it appears that technical modifications to the GON blockade—such as using a higher injectate volume to ensure more extensive spread around the nerve, and combining the procedure with concurrent preventive treatment when appropriate—may contribute to achieving faster and more effective pain control in clinical practice [19]. In our series, the larger injectate volume used in the C2 GON approach, together with its targeted anatomical location, may explain the earlier onset of pain relief observed compared to the distal technique. Moreover, although the same dose of dexamethasone (2 mg) was used in both groups, the earlier achievement of pain control in the C2 GON group resulted in fewer total injections being required. This suggests that the C2 GON technique may also reduce cumulative steroid exposure and, consequently, the risk of systemic steroid-related side effects.

No serious side effects were observed in any of the GON block procedures performed. While local alopecia and subcutaneous atrophy, which could occur 1–2 weeks post-procedure for distal GON blocks, were not encountered in our study, these effects could have been permanent and might have led to aesthetic concerns that might have discouraged patients from pursuing the block [19]. Consequently, this side effect associated with steroid use was unlikely in C2 GON blocks due to the anatomical area involved. Therefore, although there might have been no added benefit of including steroids in the treatment of chronic migraine, the significance of incorporating steroids in managing headache disorders like cluster headache supported the notion that C2 GON block had an advantage over distal GON block [14, 19].

## 5. Limitations

This study had several limitations that should be considered when interpreting the results. The sample size of 48 patients, divided evenly between the distal and C2 GON blockade groups, was relatively small and might have limited the generalizability of the findings. Since no formal sample size calculation was performed, the risk of type II error could not be excluded. Although the local anesthetic and steroid regimen was standardized, variations in patient responses might have influenced the outcomes. The study focused only on short-term effects up to six weeks, so long-term efficacy and the potential need for maintenance injections were not assessed. The lack of blinding might have introduced observer bias. Additionally, as the C2 GON block is a more invasive and technically sophisticated procedure compared to the distal block, it may have carried a higher risk of placebo response, as has been described for interventions perceived as more advanced. The risk of complications from C2 GON blockade, related to its proximity to arterial structures, was also not fully evaluated. Nevertheless, as all eligible patients were included, the findings provided valuable preliminary insights. Larger, prospective randomized trials are needed to confirm these results and to further evaluate the comparative effectiveness of distal versus C2-level GON blocks.

## 6. Conclusions

C2 GON block provided earlier and more complete relief in patients with episodic CH compared to distal GON block, with fewer total injections required. This technique may offer advantages in terms of faster clinical response, reduced steroid exposure, and lower risk of local cosmetic complications. While these results are promising, further large-scale, randomized studies are necessary to validate the comparative efficacy and safety of these approaches.

## Supplementary Material

Supplementary material associated with this article can be found, in the online version, at https://files.jofph.com/ files/article/1999376967088914432/attachment/ Supplementary%20material.docx.



## References

[1] Petersen AS, Lund N, Goadsby PJ, Belin AC, Wang SJ, Fronczek R, *et al*. Recent advances in diagnosing, managing, and understanding the pathophysiology of cluster headache. The Lancet Neurology. 2024; 23: 712–724. 10.1016/S1474-4422(24)00143-1 38876749

[2] Headache Classification Committee of the International Headache Society (IHS). The international classification of headache disorders, 3rd edition. Cephalalgia. 2018; 38: 1–211. 10.1177/0333102417738202 29368949

[3] Lund NLT, Petersen AS, Fronczek R, Tfelt-Hansen J, Belin AC, Meisingset T, *et al*. Current treatment options for cluster headache: limitations and the unmet need for better and specific treatments—a consensus article. The Journal of Headache and Pain. 2023; 24: 121. 10.1186/s10194-023-01660-8 PMC10476341 37667192

[4] Kim SA, Choi SY, Youn MS, Pozo-Rosich P, Lee MJ. Epidemiology, burden and clinical spectrum of cluster headache: a global update. Cephalalgia. 2023; 43: 3331024231201577. 10.1177/03331024231201577 37728577

[5] May A, Evers S, Goadsby PJ, Leone M, Manzoni GC, Pascual J, *et al*. European Academy of Neurology guidelines on the treatment of cluster headache. European Journal of Neurology. 2023; 30: 2955–2979. 10.1111/ene.15956 37515405

[6] San-Juan D, Velez-Jimenez K, Hoffmann J, Martínez-Mayorga AP, Melo-Carrillo A, Rodríguez-Leyva I, *et al*. Cluster headache: an update on clinical features, epidemiology, pathophysiology, diagnosis, and treatment. Frontiers in Pain Research. 2024; 5: 1373528. 10.3389/fpain.2024.1373528 PMC10957682 38524268

[7] Karaoğlan M, İnan LE. A comparison of the clinical efficacy of GON block at the C2 level and GON block at the classical distal occipital level in the treatment of migraine. Clinical Neurology and Neurosurgery. 2022; 215: 107190. 10.1016/j.clineuro.2022.107190 35286995

[8] Stern JI, Chiang CC, Kissoon NR, Robertson CE. Narrative review of peripheral nerve blocks for the management of headache. Headache. 2022; 62: 1077–1092. 10.1111/head.14385 36286600

[9] Karaoğlan M, Kılınç A. The comparative clinical effectiveness of withdrawal treatment with greater occipital nerve block at the C2 level with dexamethasone or bupivacaine in medication-overuse headache. Turkish Journal of Neurology. 2025; 31: 196–212.

[10] Brandt RB, Doesborg PGG, Meilof R, de Coo IF, Bartels E, Ferrari MD, *et al*. Repeated greater occipital nerve injections with corticosteroids in medically intractable chronic cluster headache: a retrospective study. Neurological Sciences. 2022; 43: 1267–1272. 10.1007/s10072-021-05399-5 PMC8789713 34159486

[11] Karacan Golen M, Tepe N, Işik ŞM. Effectiveness of greater occipital nerve blockade in chronic cluster headache. European Review for Medical and Pharmacological Sciences. 2024; 28: 4170–4178. 10.26355/eurrev_202408_36669 39229846

[12] Leroux E, Valade D, Taifas I, Vicaut E, Chagnon M, Roos C, *et al*. Suboccipital steroid injections for transitional treatment of patients with more than two cluster headache attacks per day: a randomised, double-blind, placebo-controlled trial. The Lancet Neurology. 2011; 10: 891–897. 10.1016/S1474-4422(11)70186-7 21903477

[13] Kupperman W, Goyal KK. Particulate *vs.* nonparticulate steroids, a discussion on safety and efficacy. Advances in Clinical Radiology. 2020; 2: 247–255.

[14] Gordon A, Roe T, Villar-Martínez MD, Moreno-Ajona D, Goadsby PJ, Hoffmann J. Effectiveness and safety profile of greater occipital nerve blockade in cluster headache: a systematic review. Journal of Neurology, Neurosurgery & Psychiatry. 2024; 95: 73–85. 10.1136/jnnp-2023-331066 36948579

[15] Ornello R, Lambru G, Caponnetto V, Frattale I, Di Felice C, Pistoia F, *et al*. Efficacy and safety of greater occipital nerve block for the treatment of cluster headache: a systematic review and meta-analysis. Expert Review of Neurotherapeutics. 2020; 20: 1157–1167. 10.1080/14737175.2020.1809379 32781922

[16] Chowdhury D, Kordcal SR, Nagane R, Duggal A. ANODYNE study: a double-blind randomized trial of greater occipital nerve block of methylprednisolone and lignocaine versus placebo as a transitional preventive treatment for episodic cluster headache. Cephalalgia. 2024; 44: 3331024241291597. 10.1177/03331024241291597 39415681

[17] Karaoğlan M. Addressing limitations of single GON blockade treatment and repetitive intervention with GON block or onabotulinum toxin A in chronic migraine-part 2 of three men in a boat study. Clinical Neurology and Neurosurgery. 2024; 240: 108242. 10.1016/j.clineuro.2024.108242 38518628

[18] Gürsoy G, Tuna HA. Comparison of two methods of greater occipital nerve block in patients with chronic migraine: ultrasound-guided and landmark-based techniques. BMC Neurology. 2024; 24: 311. 10.1186/s12883-024-03816-8 PMC11373286 39232647

[19] Kashyap PV, Chabri S. Steroids in headache: a comprehensive review of recent research. Annals of Neurosciences. 2023; 30: 256–261. 10.1177/09727531231173286 PMC10662276 38020407

