# Supplementary material for: A comparison of the clinical efficacy of GON block at the C2 level and GON block at the classical distal occipital level in the treatment of cluster headache
Source: J Oral Facial Pain Headache. 2025 Dec 12;39(4):235–41. doi: 10.22514/jofph.2025.080 (PMC12727177; doi:10.22514/jofph.2025.080)
Supplement: Supplementary file 1 [file Supplementary-material.docx]

Supplementary material

Supplementary Table 1. Baseline characteristics with statistical comparisons.

| Variable | C2 GON  (n = 24) | Distal GON  (n = 24) | Test statistic | *df* | *p*-value | Effect size |
| --- | --- | --- | --- | --- | --- | --- |
| Age (yr), mean ± SD | 38.17 ± 5.41 | 36.29 ± 5.02 | *t* (46) = 1.24 | 46 | 0.220 | 0.36 |
| Weekly attack frequency, mean ± SD | 21.54 ± 6.36 | 21.83 ± 6.02 | *t* (46) = −0.16 | 46 | 0.871 | −0.05 |
| Gender (male proportion) | 19/24 | 18/24 | χ^2^ (1, N = 48) = 0.00 | 1 | 1.000 | 0.00 |

GON: greater occipital nerve; SD: standard deviation; *df*: degrees of freedom.

Supplementary Table 2. Total number of injections with statistical comparison.

| Variable | C2 GON | Distal GON | Test statistic | *df* | *p*-value | Effect size |
| --- | --- | --- | --- | --- | --- | --- |
| Total injections | 37 | 100 | *t* (46) = −inf | 46 | <0.001 | −inf |

GON: greater occipital nerve; *df*: degrees of freedom.
